# Cannabidiol (CBD) modulates the transcriptional profile of ethanol-exposed human dermal fibroblast cells

**DOI:** 10.1007/s13353-024-00915-7

**Published:** 2024-10-28

**Authors:** Artur Gurgul, Jakub Żurowski, Tomasz Szmatoła, Mirosław Kucharski, Sebastian Sawicki, Ewelina Semik-Gurgul, Ewa Ocłoń

**Affiliations:** 1 Faculty of Veterinary Medicine, Department of Basic Sciences, University of Agriculture in Kraków, Redzina 1C, 30-248 Krakow, Poland; 2 Faculty of Animal Science, Department of Animal Physiology and Endocrinology, University of Agriculture in Kraków, Mickiewicza 24/28, 30‑059 Krakow, Poland; 3 Faculty of Animal Science, Department of Animal Reproduction, Anatomy and Genomics, University of Agriculture in Kraków, Mickiewicza 24/28, 30-059 Krakow, Poland; 4 Department of Animal Molecular Biology, National Research Institute of Animal Production, Krakowska 1, 32-083 Balice, Poland; 5 Faculty of Veterinary Medicine, Laboratory of Recombinant Proteins Production, University of Agriculture in Kraków, Rędzina 1C, 30-248 Kraków, Poland

**Keywords:** Alcohol use disorder, Cannabidiol, CBD, Ethanol, Fibroblasts, Fibrosis

## Abstract

Cannabidiol (CBD) is abundant in the *Cannabis sativa* plant and exhibits complex immunomodulatory, anxiolytic, antioxidant, and antiepileptic properties. Several studies suggest that CBD could be used for different purposes in alcohol use disorder (AUD) and alcohol-related injuries to the brain and the liver. In this study, we focused on analyzing transcriptional alterations in human dermal fibroblasts (HDFs) cell line challenged simultaneously with ethanol and CBD as an ethanol-protective agent. We aimed to expose the genes and pathways responsible for at least some of the CBD effects in those cells that can be related to the AUD. Transcriptome analysis was performed using HDFs cell line that expresses both cannabinoid receptors and can metabolize ethanol through alcohol dehydrogenase activity. Fibroblasts are also responsible for the progression of liver fibrosis, a common comorbidity in AUD. With the use of a cellular test, we found that CBD at the lowest applied concentration (0.75 μM) was able to stimulate depressed metabolism and reduce the level of apoptosis of cells treated with different concentrations of ethanol to the level observed in the control cells. Similar observations were made at the transcriptome level, in which cells treated with ethanol and CBD had similar expression profiles to the control cells. CBD also affects several genes connected with extracellular matrix formation (especially its collagen constituent), which can have potential implications for, e.g., fibrosis process.

**Supplementary Information:**

The online version contains supplementary material available at 10.1007/s13353-024-00915-7.

## Introduction

Cannabidiol (CBD) is a chemical compound belonging to the cannabinoid family, and it is a natural constituent of *Cannabis sativa* (or Indian hemp; the subfamily *Cannaboideae* of family *Moraceae*). Cannabinoids are formed through a terpene combined with resorcinol, or according to a new nomenclature, they are a benzopyran derivate (Meissner and Cascella 2022). Several other cannabinoids are present in hemp, including low amounts of well-known tetrahydrocannabinol (THC) and its psychoactive isomer delta (Δ9-THC) (Sholler et al. 2020). Since its isolation, CBD has gained interest as a medication, and it is now a registered drug in the USA for managing and treating seizure disorders, Lennox-Gastaut syndrome, and Dravet syndrome (Meissner and Cascella 2022).

In contrast to Δ9-THC which is recently considered a risk factor even for schizophrenia (Khanal et al. 2023), CBD has no psychotomimetic or hallucinogenic effects (Iseger and Bossong 2015). The confirmed pharmacological effects of CBD are associated with its actions on cognitive processes and anxiety regulation. It is being considered a possible treatment for other neuropsychiatric disorders, such as anxiety, depression, substance use disorders, and epilepsy (Campos et al. 2016; Khanal et al. 2024; Lee et al. 2017) Apart from its actions in the brain, CBD exerts systemic effects, mainly due to its complex immunomodulatory and antioxidant properties (Atalay et al. 2019; Booz 2011). This creates an opportunity for CBD application in various inflammatory and immunological disorders, and related disorders such as neurodegenerative diseases (Bhunia et al. 2022), cardiovascular diseases (Stanley et al. 2013), diabetes (Santiago et al. 2019), and even cancer (Massi et al. 2013; O’Brien 2022). One of the interesting features of CBD is that it might mediate protective effects for various aspects of alcohol use disorder (AUD) (De Ternay et al. 2019). It has been demonstrated that CBD reduces the total level of alcohol drinking in animal models, and it reduces the motivation for ethanol consumption, relapse, and anxiety induced by alcohol withdrawal (Filev et al. 2017; Gonzalez-Cuevas et al. 2018; Viudez-Martínez et al. 2018). CBD also reduces alcohol-associated liver disease manifesting through steatosis and fibrosis. This effect is achieved by modulating inflammation in the liver, reducing lipid accumulation and oxidative stress, and by stimulating the death of activated hepatic stellate cells (Lim et al. 2011; Wang et al. 2017; Yang et al. 2014). CBD also decreases alcohol-induced brain damage, due to its neuroprotective abilities, preventing neuronal death through its antioxidant and immunomodulatory features (De Ternay et al. 2019; Hamelink et al. 2005; Lees et al. 2021; Liput et al. 2013; Magen et al. 2009; Stavro et al. 2013).

The molecular mechanisms of CBD action are exceptionally complex, and thus they are not yet fully elucidated. It has been shown that CBD has only a weak affinity to the cannabinoid receptors CB1 and CB2, and it is rather considered their noncompetitive, negative allosteric modulator (Pertwee 2008; Tham et al. 2019). CBD is also a partial agonist of the serotonin 5-HT1A receptor, and an allosteric modulator of the opioid receptors mu and delta (Kathmann et al. 2006). Furthermore, CBD is a partial antagonist of G protein–coupled receptor 55 (GRP55) (Ryberg et al. 2007), which could be involved in the decrease in neuronal excitability, by modulating gamma-aminobutyric acid-ergic (GABAergic) neurotransmission (Chen et al. 2018; Musella et al. 2017). The other known mechanism of CBD action involves the inhibition of anandamide hydrolysis (which is a part of the endocannabinoid system) via inhibiting the activity of fatty acid amide hydrolase (FAAH) (Deutsch 2016; Leweke et al. 2012). CBD was also shown to activate peroxisome proliferator–activated receptor γ (PPAR-γ) (Devinsky et al. 2014), and was identified as a positive allosteric modulator of serotonin 1A receptors (5-HT1A receptors) (Rock et al. 2012). Moreover, CBD is able to activate the transient receptor potential vanilloid type 1 (TRPV1) (Anand et al. 2020) and regulate calcium (Ca^2+^) homeostasis by acting on low-voltage-activated (T-type) Ca^2+^ channels, thus modulating cellular calcium intake (Ryan et al. 2009). The mechanisms of the systemic antioxidant and the immunomodulatory properties of CBD seem to be even more complex. It has been shown that it interferes with several inflammation-related pathways, among which are the nuclear factor kappa-light-chain-enhancer of activated B cells (NF-κB) pathway (Khaksar and Bigdeli 2017), as well as the interferon β/signal transducer and the activator of transcription proteins (IFNβ/STAT) pathway (Juknat et al. 2012). CBD can also modulate the activities of immune cells (neutrophils, macrophages, and T cells), and it reduces the synthesis of inflammatory mediators such as, e.g., interferon-c (IFN-c), interferon-γ (IFN-γ) (Lee et al. 2016), tumor necrosis factor α (TNF-α), interleukin-1β (IL-1β), and -6 (IL-6) (Lee et al. 2016; Pazos et al. 2013; Wang et al. 2017).

Due to those various effects of CBD on nervous system functioning and the courses of inflammation processes, CBD has a large potential as a complementary therapeutic in AUD (De Ternay et al. 2019). Its main therapeutic effect in AUD may be connected with neuroprotective activities (mainly antioxidant) and the reduction in inflammation that arises in the liver as a result of alcohol abuse. CBD also has the potential to counteract ethanol-induced cell toxicity by modifying numerous cellular pathways whose alterations may be detected at the molecular and transcriptomic levels.

Until now, only a limited number of studies have analyzed the effects of CBD on cell or organ transcriptomes. The currently available reports showed CBD effects in myelin oligodendrocyte glycoprotein (MOG)–sensitized lymphocytes (Yang et al. 2019), microglial cells (Juknat et al. 2012), keratinocytes (Casares et al. 2020), human Sertoli cells (Li et al. 2022), macrophages (Tomer et al. 2022b), cGAMP-stimulated THP1 cells treated with vehicle/CBD (Tomer et al. 2022a), zebrafish (*Danio rerio*) larvae (Pandelides et al. 2021), A549-ACE2 cells infected with SARS-CoV-2 (Nguyen et al. 2023), and APP/PS1 mice hippocampuses (Hao and Feng 2021), using a microarray approach or RNA sequencing (RNA-Seq). One particular study analyzed changes in a CBD oil extract–treated fibroblast transcriptome after induced fibrosis, which is a common disorder in the livers of alcohol-abusing patients. The study showed that CBD-rich cannabis extracts downregulated the expression of several key fibrotic genes, indicating their anti-fibrotic potential (Pryimak 2021). Nevertheless, according to our best knowledge, there is no report on the global transcriptional changes in cells challenged simultaneously with ethanol and CBD as an ethanol-protective agent. Due to the applied high-throughput transcriptome analysis method, we could only make a simplified hypothesis—that CBD will alter the cell transcriptome, exposing the genes and pathways responsible for at least some of the CBD therapeutic effects. We also assumed that CBD will (to some extent) prevent adverse transcriptional changes driven by alcohol administration, and that this effect will be detectable at the transcriptome level. As a by-product of this analysis, ethanol-stimulated transcriptome changes were also described. We moreover considered that the CBD effects are dose- and time-dependent. As a model for these analyses, we selected the human fibroblast cell line since it is the most abundant cell type in the human body that expresses both cannabinoid receptors (CB1 and CB2) (McPartland 2008), and that is able to metabolize ethanol through alcohol dehydrogenase (ADH) activity (Petersen et al. 1980). Furthermore, fibroblasts or fibroblast-like cells are one of the most important cell types that are responsible for the progression of liver fibrosis, a common disorder in AUD (Rhodes et al. 2021; Zhang et al. 2016). Therefore, the objective of this study was to assess the protective effects of CBD against the ethanol-mediated toxic effects on human dermal fibroblasts (HDF) that are relevant to fibrosis.

## Material and methods

### Cell cultures

The human dermal fibroblasts (HDFs) cell line was obtained from PromoCell GmbH (Cat. No. C-12302, Germany). HDFs were maintained in DMEM with GlutaMAX (Thermo Fisher Scientific, USA) medium supplemented with 10% heat-inactivated FBS and 1% Gibco® Antibiotic–Antimycotic Solution (10,000 U/mL penicillin, 10,000 µg/mL streptomycin, and 25 µg/mL Amphotericin B; Thermo Fisher Scientific, USA). Cultures were seeded into flasks containing supplemented medium and maintained at 37° C in a humidified atmosphere of 5% CO_2_ and 95% air.

### Experimental design and treatment

To analyze the effects of pure CBD on cell metabolism and apoptotic processes, cells were incubated in a culture medium containing 0.75, 1.5, and 3 µM of CBD (MERCK, USA) solution. The CBD was dissolved in DMSO (a final DMSO concentration of 0.1% in the assay media for all assays). Therefore, the cells were separately treated with 0.1% DMSO in the DMEM, with GlutaMAX supplemented with 10% FBS as a vehicle control. Additionally, to evaluate the effects of ethanol alone, three ethanol concentrations were applied in culture medium, corresponding to 0.15, 0.25, and 0.75% EtOH. For an analysis of the ethanol-protective effects of CBD, two concentrations of ethanol were applied (namely, 0.25 and 0.75%) in the culture medium, and these were combined with all three tested concentrations of CBD (0.75, 1.5, and 3 µM). In all of the experiment setups, cells were analyzed after 6, 12, and 24 h of incubation. All treatments were performed in triplicate to capture the possible technical variations that may occur at all stages of the experiment. CBD concentrations were selected based on previous literature reports describing experiments on in vitro cultured cells (Kim et al. 2021). Ethanol concentrations were selected based on previous reports to reflect different levels of alcoholic intoxication (Casañas-Sánchez et al. 2016; Kar et al. 2021). It was shown, that 10–100 mM ethanol range is considered physiological and 25 mM ethanol being close to 0.08% blood alcohol level achieved in vivo after 4–5 drink equivalents (Dolganiuc and Szabo 2009). In this study, the applied ethanol concentrations were in the range of 32–163 mM, so corresponding rather to acute alcohol consumption. For transcriptome analysis, only treatments with the robust and the most distinct effects on the MTT assay results were selected, namely, extreme CBD concentrations (of 0.75 and 3 μM) and ethanol concentrations (0.25 and 0.75%). The transcriptome analysis focused only on cells treated for 6 and 12 h.

### The MTT and caspase-Glo 3/7 assays

#### The MTT assay

Cell viability was measured via a quantitative colorimetric assay with MTT according to the manufacturer (Thermo Fisher Scientific, USA) (Mosmann 1983). Briefly, 10 µL of the 12 mM MTT stock solution was added to 100 µL of medium in each well, and the plates were placed in a humidified incubator at 37 °C with 5% CO_2_ and 95% air for 4 h. Metabolically active cells convert the yellow MTT tetrazolium compound to a purple formazan product. The insoluble formazan was dissolved with SDS–HCl solution; and the colorimetric determination of MTT reduction was measured with a TEKAN Infinite M200 PRO microplate reader at 570 nm. Control cells treated with DMEM-GlutaMAX were taken as 100% viability.

#### Caspase-Glo 3/7 assay

To evaluate the activities of two key caspases of apoptosis—caspases 3 and 7, the caspase-Glo 3/7 luminescent assay (Promega, Germany) was employed. Fibroblasts were grown in a 96-well white plate and exposed to CBD and ethanol as previously described. Then, 100 μL of the caspase-Glo 3/7 reagent was added to each well. The plates were incubated for 2 h in the dark at room temperature. The addition of the caspase-Glo 3/7 reagent results in cell lysis, followed by the caspase cleavage of the substrate (containing the tetrapeptide sequence DEVD) and the generation of a luminescent signal produced by luciferase. Luminescence intensity was determined with a TEKAN Infinite M200 PRO microplate reader at 570 nm. Relative caspase 3/7 activity was normalized to the control group.

### 3’ mRNA-Seq analysis

Following treatment, cells were trypsinized, transferred to 1.5-mL tubes, and centrifuged at 500 RCF for 7 min to form pellets. Supernatants were immediately removed, and cells were snap-frozen at − 80 °C until RNA isolation. Total RNA was isolated using a standard TRI Reagent™ Solution (ThermoFisher Scientific, USA) procedure and evaluated for quality using the TapeStation 4150 System (Agilent, USA). RNA quantification was performed using the Qubit RNA BR assay (ThermoFisher Scientific, USA). In total, 50 ng of RNA was taken to the library preparation process with the QuantSeq 3′ mRNA-Seq Library Prep Kit FWD (Lexogen, Austria). QuantSeq generates only one fragment per transcript at the 3′ end instead of covering the full length of the transcript with reads, as in conventional mRNA-Seq. This allows for a reduced number of raw reads generated per sample to about 3 million (M), and the procedure is highly resistant to alterations in RNA quality, being efficient even for FFPE samples. The obtained indexed libraries were controlled for quality using the TapeStation4150 System (Agilent, USA) with D1000 ScreenTapes, and quantified using the Qubit dsDNA BR kit (ThermoFisher Scientific, USA). Equimolar pools of libraries were finally sequenced in a single-end 75 bp run on a NextSeq 550 System (Illumina, USA) to obtain at least 5 M of reads per library. Raw reads, as well as raw read counts, were deposited in the Gene Expression Omnibus (GEO) and Sequence Read Archive (SRA) databases from the National Center for Biotechnology Information (NCBI) under the accession number GSE232624.

### Sequencing data analysis

Raw reads were controlled for quality using FastQC (v0.11.9) software. Then, trimming and filtering steps were taken using Flexbar software (3.5.0) (Dodt et al. 2012) to remove low-quality read ends, adapter sequences, and reads that were too short after trimming. Filtered reads were mapped to the GRCh38 (hg38) human genome assembly with STAR aligner software (2.7.5c) (Dobin et al. 2013). The mapped reads were counted within annotation 108 (Ensembl) features using Htseq-count (1.99.2) (Anders et al. 2015) software. Reads normalization and differential expression (DE) analysis were performed using DESeq2 (v3.16) (Love et al. 2014) software. The genes detected as DE (FDR < 0.05) were analyzed for their function and enrichment in a specific GO (biological processes) and Panther pathways categories using WebGestalt (WEB-based Gene SeT AnaLysis Toolkit) (Liao et al. 2019). Additional functional enrichment analysis was performed with GeneMANIA software (Warde-Farley et al. 2010). Venn diagrams were prepared using online tools (https://bioinformatics.psb.ugent.be/webtools/Venn/) (Heberle et al. 2015).

### qPCR validation of 3′ mRNA-Seq results

For the validation of RNA-Seq results with the qPCR method, three genes were selected among the ones that were affected by the CBD and ethanol treatments. The first was profilin 1 (*PFN1*; F-5′-acgtgaatgggctgacactt-3′; R-5′-agtgacattgaaggtggggg-3′), which was upregulated by CBD, and the second was collagen type VIII alpha 1 chain gene (*COL8A1*; F-5-gctggtgcctactatgggatc-3′; R-5′-caggggctggtattgtggaa-3′), which was downregulated as a result of different CBD treatments. The last one was AKT serine/threonine kinase 2 (*AKT2*; F-5′-gcctcttcgagctcatcctc-3′; R-5′-tccttcttaagcagcccagc-3′), which was affected by at least three different CBD/EtOH treatments. qPCR was performed for 21 samples (belonging to nine treatment groups—2–3 samples per each), for which a sufficient amount of RNA was left after transcriptome analysis. Reverse transcription PCR (RT-PCR) was performed using 0.2 μg of total RNA for each sample using a High Capacity cDNA Reverse Transcription Kit (Thermo Fisher Scientific, USA) according to the manufacturer’s protocol. Gene expression levels were quantified using the qPCR method. Briefly, the expression of the genes was analyzed using a QuantStudio™ 3 Real-Time PCR System (Thermo Fisher Scientific, USA) with PowerUp™ SYBR™ Green Master Mix (Thermo Fisher Scientific, USA), as recommended by the manufacturer’s protocol. Target gene expression levels were normalized to glyceraldehyde-3-phosphate dehydrogenase (*GAPDH*; F-5′-caccatcttccaggagcgag-3′; R-5′-agagggggcagagatgatga-3′) and 18S ribosomal RNA (18S rRNA; F-5′-tttctcgattccgtgggtgg-3′; R-5′-tcaatctcgggtggctgaac-3′). The individual gene expression level was calculated via relative quantitative (RQ) analysis and the Pfaffl model, which included the reaction efficiency for individual genes (Pfaffl 2001). The data compliance among the qPCR and RNA-Seq methods was assessed via correlation coefficient analysis using JASP statistical software (https://jasp-stats.org/).

### Statistical analysis

Statistical analysis was performed using JASP v0.17.1 software (https://jasp-stats.org/). First, the data distribution was evaluated using the Shapiro–Wilk test, and the variance equality was tested with the Levene test. In the case of data with a normal distribution and equal variances, the parametric one-way ANOVA test was used, and comparisons between the groups were analyzed with the Tukey test. In the case of data not having a normal distribution, or if their variances differed statistically significantly, the Kruskal–Wallis test (being a non-parametric equivalent of the ANOVA test) was used. Comparisons between the groups were analyzed with the Dunn test.

## Results

### Effect of CBD treatment on cell viability and apoptosis

**Fig. 1:**
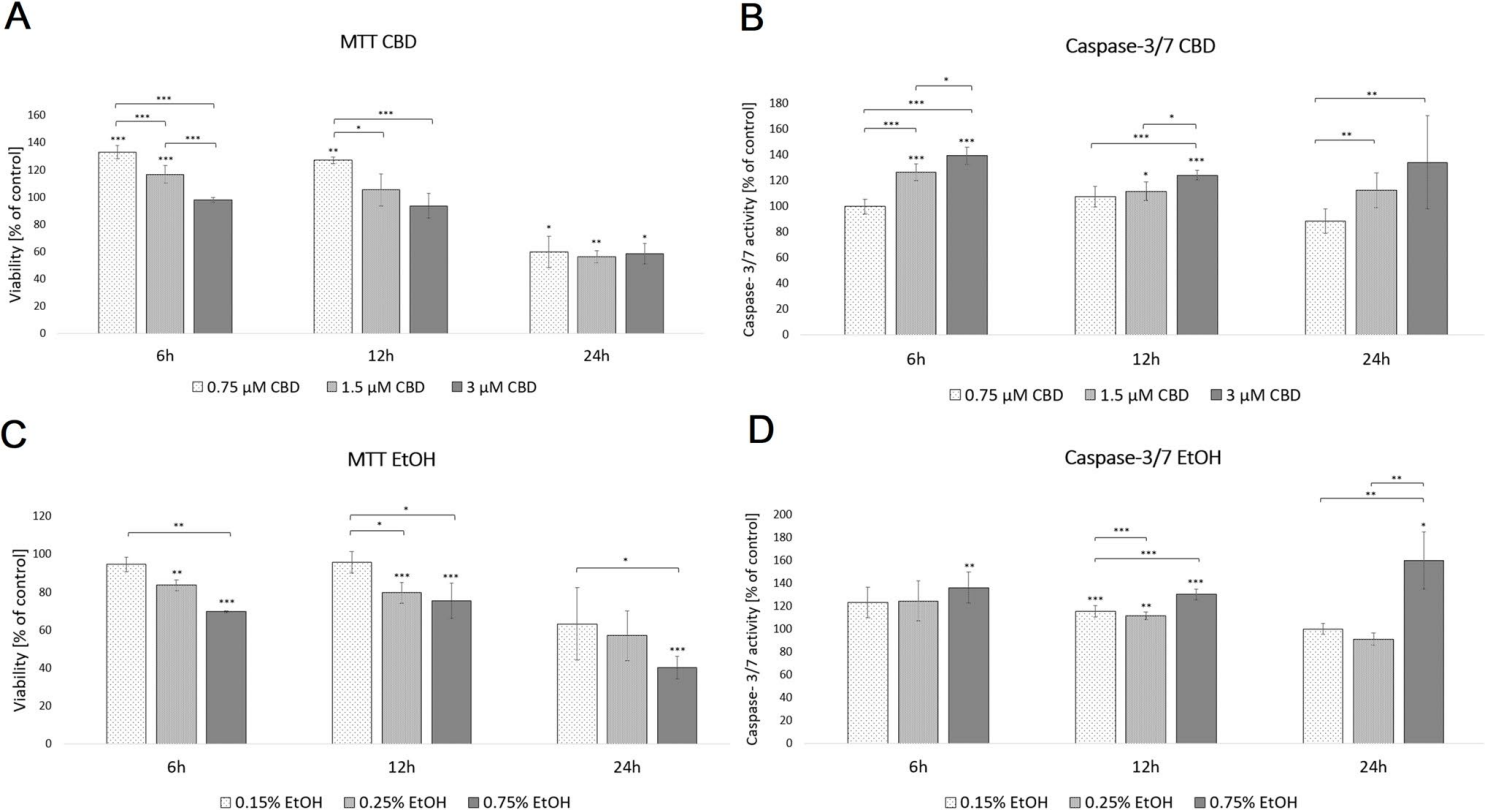
Effect of supplementation with different dosages of CBD (**A**, **B**) and ethanol (**C**, D) on HDF metabolic activity (**A**, **C**) and apoptosis (**B**, **D**) measured via MTT and caspase 3/7 assays. *Statistically significant differences at *p* < 0.05; **statistically significant differences at *p* < 0.01; ***statistically significant differences at *p* < 0.001. Asterisks just above deviation bar show significance relative to control cells (set as 100%)

In order to evaluate cell viability, we used the MTT assay. The results indicate that cell incubation for 6 h with media supplemented with 0.75 or 1.5 µM CBD determined a 33% and 16% increase in cell survival (*p* < 0.001 compared to untreated cells), while higher concentrations (3 µM CBD) did not significantly affect the HDF viability. A 12-h incubation period with CBD increased cell viability (~ 27%) only at a 0.75 µM CBD concentration (*p* < 0.01). Interestingly, exposure to CBD for 24 h significantly decreased cell survival by 40.1% (0.75 µM CBD, *p* < 0.05), 43.9% (1.5 µM CBD, *p* < 0.01), and 41.5% (3 µM CBD, *p* < 0.05), respectively (Fig. 1; Supplementary File 1).

To assess the effects of CBD on the activities of the two key caspases of apoptosis (caspase 3/7), we employed the cell-based homogeneous caspase-Glo assay kit. The results showed that after treatment with 1.5 and 3 µM CBD for 6 and 12 h, HDF-cellular caspase 3/7 activities significantly increased (Fig. 1; Supplementary File 1). Additionally, only the higher concentration of CBD activated caspase 3/7 in HDF cells after 24 h incubation (*p* < 0.05).

### Sequencing reads statistics

**Fig. 2:**
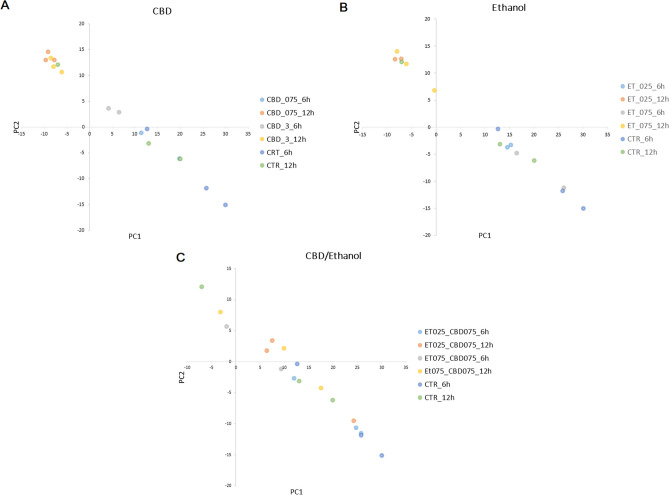
Principal component analysis of all gene expression in the studied cells. **A** CBD treatment; **B** ethanol treatment; **C** CBD/ethanol co-treatment

In this study, we generated sequencing reads for 42 cell cultures belonging to 14 treatment groups. For six libraries, the number of sequencing reads was insufficient and was thus excluded from further analysis. In total, for the remaining libraries, we generated 243.6 million (M) 75 bp single-end sequencing reads (6.8 M on average per sample; Supplementary File 2). After initial filtering, 2.8 M reads on average per sample were uniquely mapped against the reference genome, of which 66.7% was mapped to annotation features. The general sample expression profile showed clear transcriptional changes among the treatment groups and controls, with more visible alterations for higher ethanol and CBD concentrations, and longer incubation times, as shown through principal component analysis (PCA) using all genes (Fig. 2).

### Effect of CBD on gene expression profile differentiation in fibroblasts

**Fig. 3:**
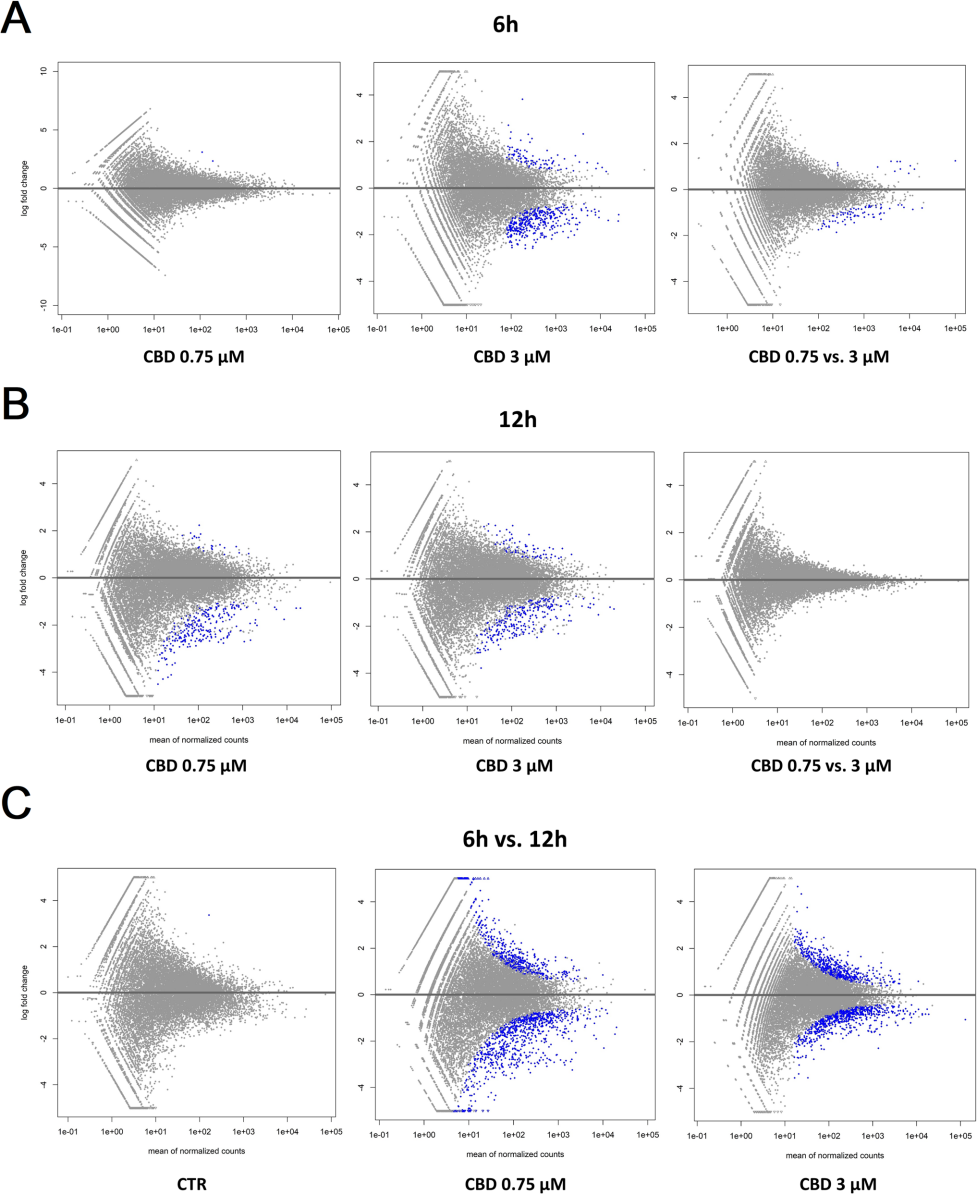
MA plots for changes in transcriptome after CBD treatment. **A** Treatment for 6 h; **B** treatment for 12 h; **C** comparison of 6 h and 12 h treatments. Treatments are compared against control samples unless specified differently in the plot

**Fig. 4:**
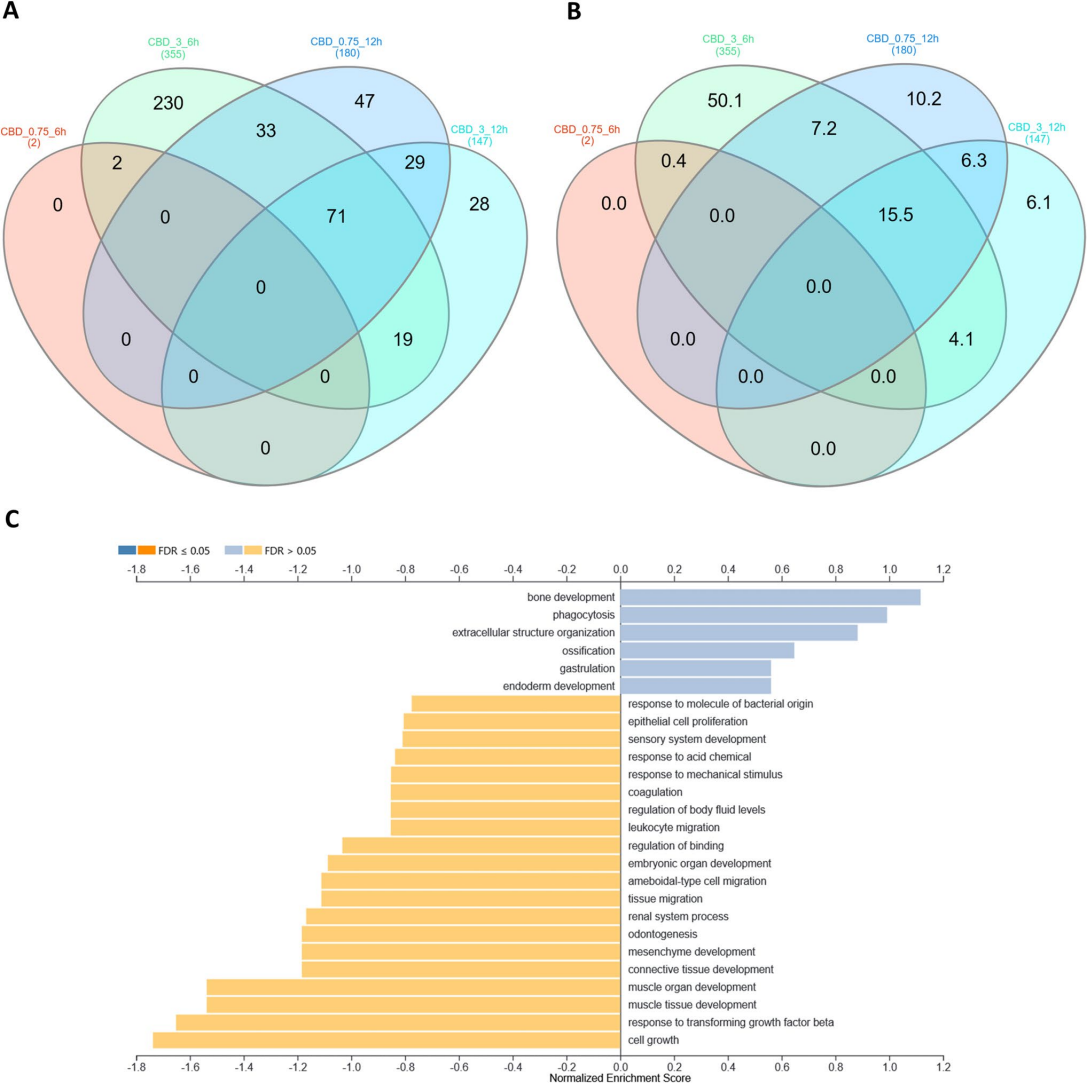
Comparative and functional analyses of genes affected by CBD treatment. **A** Venn diagram for genes affected by different CBD dosages and times of incubation; **B** Venn diagram for genes affected by different CBD dosages and times of incubation—percentage share; **C** Gene Set Enrichment Analysis (GSEA) in GO biological processes for 71 genes common for different CBD treatments

Changes in the expression profiles of fibroblasts following CBD treatment were evaluated based on several pairwise comparisons of RNA-Seq results for the cells treated with different concentrations/incubation times against the control group, as well as among different treatments (Fig. 3; Supplementary File 3). This analysis showed that CBD affects gene expression in both dosage- and time-dependent manners (Fig. 3); however, no significant effects of CBD concentrations at 12-h treatment were detected. Furthermore, no gene expression changes were detected for the control cells at both the analyzed time points. CBD mainly caused the downregulation of gene expression (Table 1), and this effect was stronger for cells treated for 12 h and/or with higher dosages of CBD. Comparative analysis revealed a panel of 71 genes (Fig. 4; Supplementary File 4) that were altered in all CBD treatments, except for 0.75 µM CBD for 6 h, which had only a very minor effect on gene expression. In this treatment, which had the strongest positive effect on cell metabolic activity in the MTT assay, only two genes were upregulated, namely *PFN1* and *C1R*. Those genes were altered only after 6 h of incubation with both the applied CBD concentrations, but not in cells treated for 12 h. The genes affected by most of the CBD treatments were poorly annotated, and only 29 of them had records in the databases used. All of the genes but one (*RPS18*) were downregulated through CBD treatment. The genes did not enrich significantly after multiple testing correction of any of the analyzed GO categories or pathways. On a pointwise level, however, processes such as muscle development and the response to transforming growth factor-beta and cell growth were significantly enriched (*p* < 0.05) (Fig. 4). As for the Panther pathways, only the integrin signaling pathway was significantly enriched (*p* < 0.05), and a statistical trend (*p* = 0.08) was observed for the enrichment of the TGF-beta signaling pathway. The other interesting pathways with high enrichment ratios were the insulin/IGF pathway-mitogen activated protein kinase kinase/MAP kinase cascade, the insulin/IGF pathway-protein kinase B signaling cascade, inflammation mediated by the chemokine and cytokine signaling pathways, and the endothelin signaling pathway and the Ras Pathway (Table 2; Supplementary File 5). An analysis of the expression networks with GeneMANIA software showed that the recognized genes formed loose and scattered expression networks. However, network-based overrepresentation analysis showed that the genes that were highly significantly enriched (FDR < 0.01) networks connected with a complex of collagen trimers and others, and connected to collagen trimers and fibrils, and growth factor binding (Supplementary File 5).

**Table 1:** Statistics on genes affected by CBD treatment (FDR < 0.05) with respect to control cells

Treatment	Incubation time (h)	Altered genes (No)	Upregulated (No/%)	Downregulated (No/%)
CBD 0.75 µM	6	2	2 (100)	0 (0)
CBD 3 µM	6	355	75 (21)	280 (79)
CBD 0.75 µM	12	180	10 (5.5)	170 (94.5)
CBD 3 µM	12	147	21 (14)	126 (86)

**Table 2:** Top ten enriched biological processes (GSEA analysis) and Panther pathways (ORA) by genes affected in most of the CBD treatments

GSEA in GO biological processes						
GO	Description	Enrichment Score	Normalized Enrichment Score	*p* value	FDR	Genes
GO:0007517	Muscle organ development	− 0.731	− 1.537	0.046	0.460	ENSG00000078401; ENSG00000125378; ENSG00000187957
GO:0060537	Muscle tissue development	− 0.731	− 1.537	0.046	0.460	ENSG00000078401; ENSG00000125378; ENSG00000187957
GO:0071559	Response to transforming growth factor-beta	− 0.784	− 1.652	0.028	0.511	ENSG00000078401; ENSG00000170801
GO:0016049	Cell growth	− 0.808	− 1.737	0.011	0.589	ENSG00000078401; ENSG00000170801; ENSG00000188375
GO:0003014	Renal system process	− 0.549	− 1.167	0.251	0.642	ENSG00000078401; ENSG00000125378
GO:0001667	Ameboidal-type cell migration	− 0.532	− 1.111	0.329	0.666	ENSG00000078401; ENSG00000125378
GO:0090130	Tissue migration	− 0.532	− 1.111	0.329	0.666	ENSG00000078401; ENSG00000125378
GO:0042476	Odontogenesis	− 0.575	− 1.184	0.281	0.666	ENSG00000078401; ENSG00000125378
GO:0060485	Mesenchyme development	− 0.575	− 1.184	0.281	0.666	ENSG00000078401; ENSG00000125378
GO:0061448	Connective tissue development	− 0.575	− 1.184	0.281	0.666	ENSG00000078401; ENSG00000125378
ORA in Panther pathways						
Pathway	Description	Expected	Enrichment Ratio	*p* value	FDR	Genes
P00034	Integrin signaling pathway	0.879	4.550	0.009	1.000	ENSG00000144810; ENSG00000171812; ENSG00000108821; ENSG00000204262
P00052	TGF-beta signaling pathway	0.482	4.150	0.081	1.000	ENSG00000130522; ENSG00000125378
P00017	DNA replication	0.101	9.939	0.096	1.000	ENSG00000188375
P00032	Insulin/IGF pathway-mitogen activated protein kinase kinase/MAP kinase cascade	0.154	6.511	0.144	1.000	ENSG00000140443
P00033	Insulin/IGF pathway-protein kinase B signaling cascade	0.185	5.395	0.171	1.000	ENSG00000140443
P00031	Inflammation mediated by chemokine and cytokine signaling pathway	1.059	1.888	0.287	1.000	ENSG00000100345; ENSG00000130522
P04393	Ras Pathway	0.371	2.698	0.314	1.000	ENSG00000129219
P00016	Cytoskeletal regulation by Rho GTPase	0.376	2.660	0.318	1.000	ENSG00000100345
P00019	Endothelin signaling pathway	0.408	2.452	0.340	1.000	ENSG00000078401
P00049	Parkinson disease	0.471	2.122	0.382	1.000	ENSG00000129219

### Effects of ethanol treatment on cell viability and apoptosis

The MTT assay of HDFs exposed to EtOH at increasing concentrations (0.15%, 0.25%, and 0.75%) for 6 and 12 h showed a significant decrease in cell survival in a concentration-dependent manner, compared with the control group (Fig. 1; Supplementary File 1). The results also demonstrated that the 24-h incubation period with EtOH reduced cell viability only at the higher concentration (*p* < 0.001).

In the caspase 3/7 assay, the luminescence levels after 12 h of exposure by HDFs to EtOH were significantly (*p* < 0.05) higher at all EtOH concentrations than those of the control group (Fig. 1; Supplementary File 1). Additionally, in the HDFs treated with 0.75% EtOH during the 6- and 24-h incubation periods, the caspase 3/7 activity levels were also increased by 35.9% (*p* < 0.01) and by 59.6% (*p* < 0.05), respectively.

### Effect of ethanol supplementation on cell transcriptomes

**Fig. 5:**
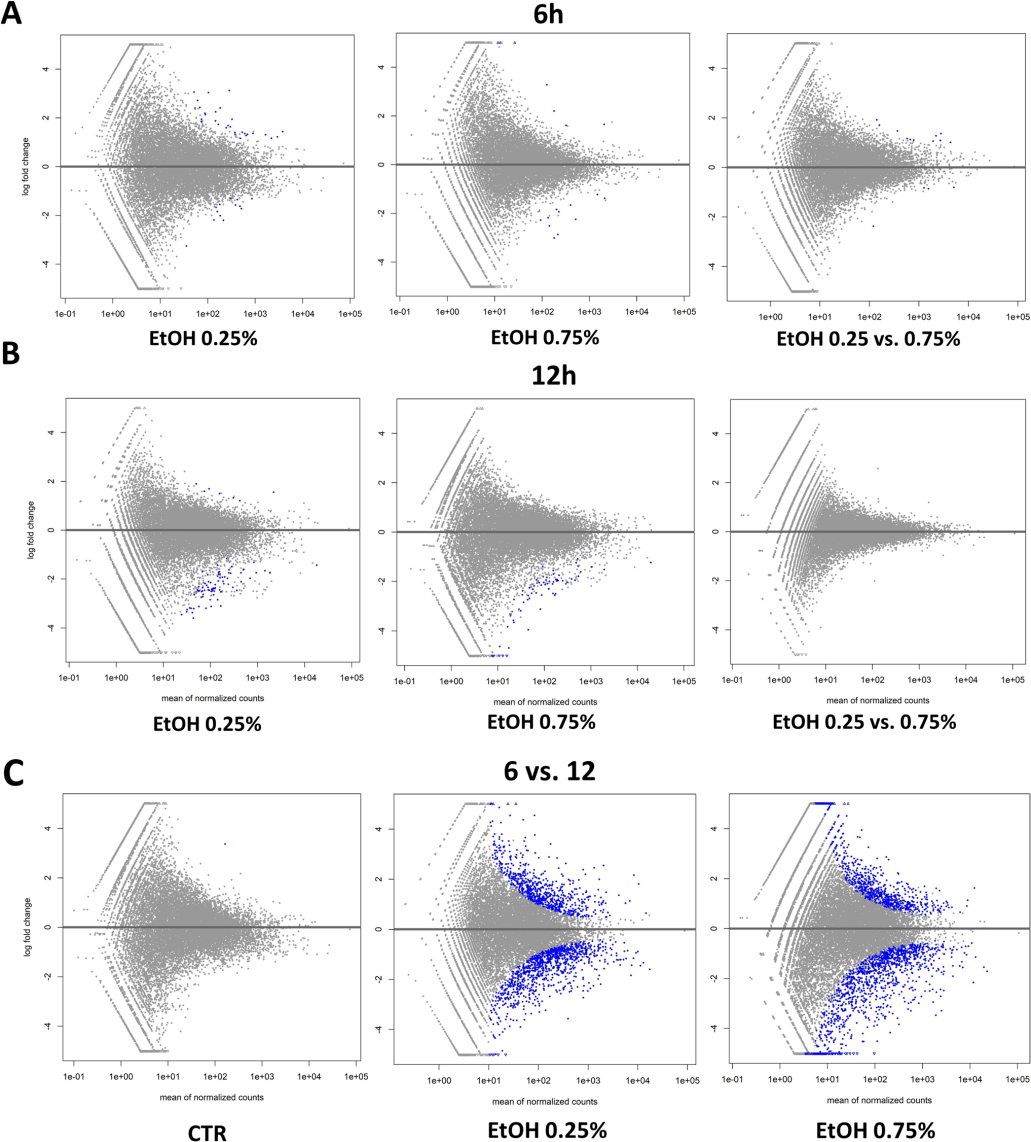
MA plots for changes in transcriptome after ethanol treatment. **A** Treatment for 6 h; **B** treatment for 12 h; **C** comparison of 6 h and 12 h treatments. Treatments are compared against control samples unless specified differently in the plot

**Fig. 6:**
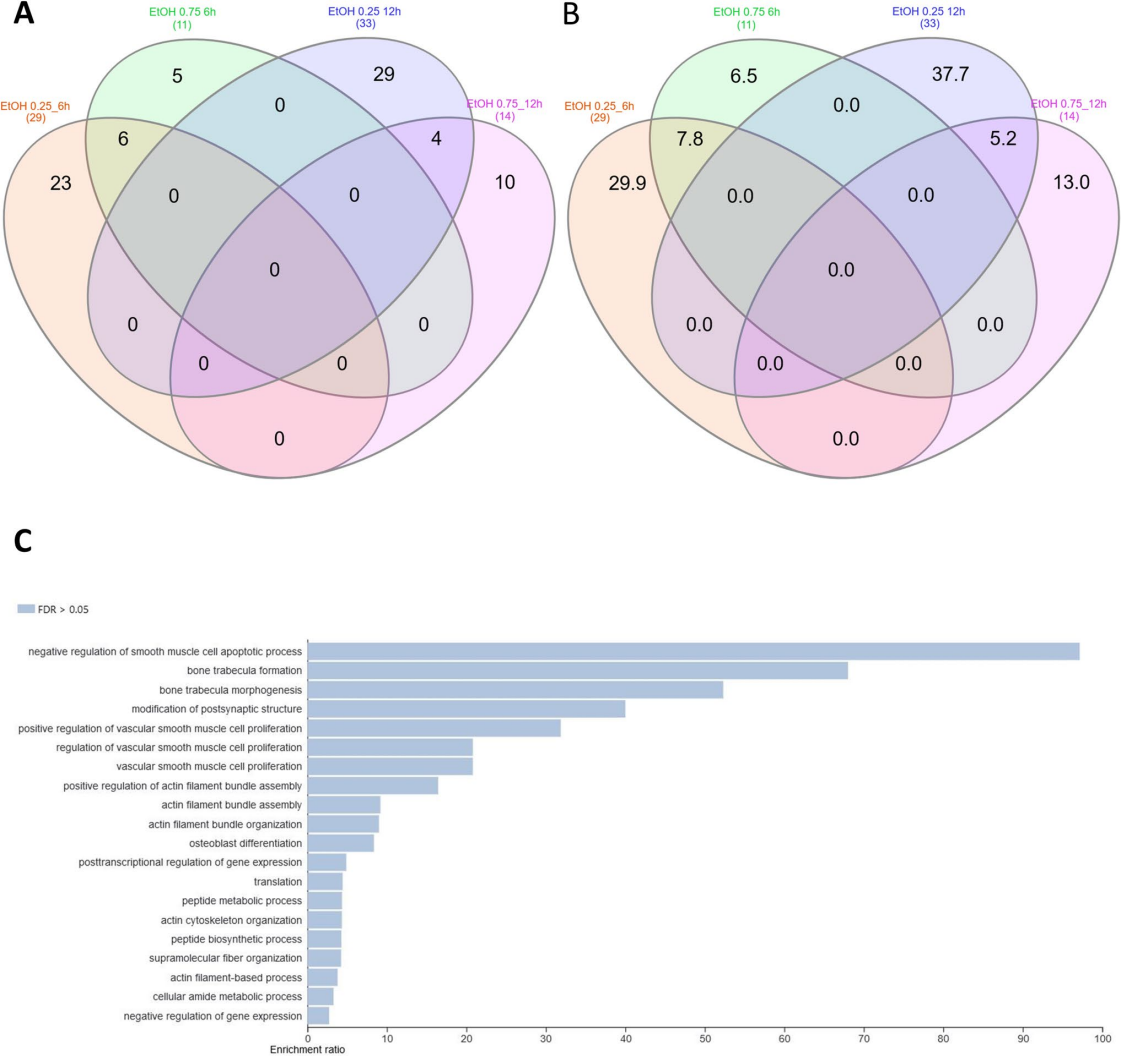
Comparative and functional analyses of genes affected by ethanol treatment. **A** Venn diagram for genes affected by different ethanol dosages and times of incubation; **B** Venn diagram for genes affected by different ethanol dosages and times of incubation—percentage share; **C** overrepresentation analysis (ORA) in GO biological processes for genes common for different ethanol treatments

The effects of the different concentrations and times of treatment with ethanol on the cell transcriptomes were evaluated in the same manner as in the case of CBD treatment, based on serial pairwise comparisons against the control and among treatments. The ethanol treatment affected the cell transcriptome in time, but to a lesser extent, in a dose-dependent manner (Fig. 5; Supplementary File 6). In fact, a higher ethanol concentration seemed to affect fewer genes than the lower applied doses (Table 3). A shorter time of EtOH treatment was mainly associated with gene upregulation, while with a longer incubation time, it was mainly the downregulation of gene expression that was observed (Table 3). Comparative analysis showed that commonly affected genes can be only found between doses at the 6-h treatment (six genes) and the 12-h treatment (four genes) (Fig. 6; Supplementary File 7). Because of this, for functional analysis, the genes affected by all of the applied ethanol treatments were used. No significant enrichments of annotation categories after correction for multiple testing were detected (Table 4; Supplementary File 8). However, point overrepresentations were found for several biological processes, including inter alia: peptide metabolic processes, the positive and negative regulation of vascular smooth muscle cell proliferation, osteoblast differentiation, bone trabecula formation, actin filament bundle assembly and organization, and the translation or posttranscriptional regulation of gene expression (*p* < 0.05). Among the genes, we also found ones that were associated with the integrin signaling pathway (*COL8A1* and *COL1A1*), cytoskeletal regulation by Rho GTPase (*PFN1*), the endothelin signaling pathway (*EDN1*), and the Wnt signaling pathway (*EDN1* and *EN1*).

**Table 3:** Statistics on genes affected by ethanol treatment (FDR < 0.05) with respect to control cells

Treatment	Incubation time (h)	Altered genes (No)	Upregulated (No/%)	Downregulated (No/%)
EtOH 0.25%	6	29	23 (79)	6 (21)
EtOH 0.27%	6	11	4 (36)	7 (64)
EtOH 0.25%	12	33	2 (7)	31 (93)
EtOH 0.27%	12	14	0 (0)	14 (100)

**Table 4:** Top ten (if available) enriched biological processes and Panther pathways (ORA analysis) by genes affected in all ethanol treatments

ORA in GO biological processes						
GO	Description	Expected	Enrichment Ratio	*p* value	FDR	Genes
GO:0006518	Peptide metabolic process	2.293	4.361	0.000	0.357	ENSG00000115461; ENSG00000172403; ENSG00000167526; ENSG00000115053; ENSG00000090621; ENSG00000156976; ENSG00000102317; ENSG00000177600; ENSG00000124766; ENSG00000104687
GO:1,904,707	Positive regulation of vascular smooth muscle cell proliferation	0.094	31.886	0.000	0.357	ENSG00000130816; ENSG00000115461; ENSG00000078401
GO:0034392	Negative regulation of smooth muscle cell apoptotic process	0.021	97.178	0.000	0.357	ENSG00000130816; ENSG00000078401
GO:0001649	Osteoblast differentiation	0.597	8.377	0.000	0.357	ENSG00000115461; ENSG00000138829; ENSG00000147274; ENSG00000196923; ENSG00000108821
GO:0010629	Negative regulation of gene expression	5.098	2.746	0.000	0.357	ENSG00000105258; ENSG00000130816; ENSG00000115461; ENSG00000167526; ENSG00000115053; ENSG00000090621; ENSG00000163565; ENSG00000102317; ENSG00000147274; ENSG00000177600; ENSG00000078401; ENSG00000179222; ENSG00000104973; ENSG00000163064
GO:0060346	Bone trabecula formation	0.029	68.024	0.000	0.357	ENSG00000138829; ENSG00000108821
GO:0006412	Translation	1.802	4.439	0.000	0.357	ENSG00000115461; ENSG00000167526; ENSG00000115053; ENSG00000090621; ENSG00000156976; ENSG00000102317; ENSG00000177600; ENSG00000124766
GO:1,904,705	Regulation of vascular smooth muscle cell proliferation	0.144	20.824	0.000	0.357	ENSG00000130816; ENSG00000115461; ENSG00000078401
GO:1,990,874	Vascular smooth muscle cell proliferation	0.144	20.824	0.000	0.357	ENSG00000130816; ENSG00000115461; ENSG00000078401
GO:0030036	Actin cytoskeleton organization	1.841	**4.347**	0.000	0.357	ENSG00000108518; ENSG00000164292; ENSG00000172403; ENSG00000166598; ENSG00000196923; ENSG00000078401; ENSG00000171345; ENSG00000171992
ORA in Panther pathways						
Pathway	Description	Expected	Enrichment Ratio	*p* value	FDR	Genes
P00017	DNA replication	0.059	17.038	0.057	1.000	ENSG00000188375
P00034	Integrin signaling pathway	0.513	3.900	0.088	1.000	ENSG00000144810; ENSG00000108821
P00016	Cytoskeletal regulation by Rho GTPase	0.219	4.559	0.200	1.000	ENSG00000108518
P00019	Endothelin signaling pathway	0.238	4.204	0.215	1.000	ENSG00000078401
P00057	Wnt signaling pathway	0.908	2.202	0.227	1.000	ENSG00000078401; ENSG00000163064
P00049	Parkinson disease	0.275	3.637	0.245	1.000	ENSG00000277791

### Effects of CBD on HDF cell viability and caspase 3/7 activity following exposure to ethanol

**Fig. 7:**
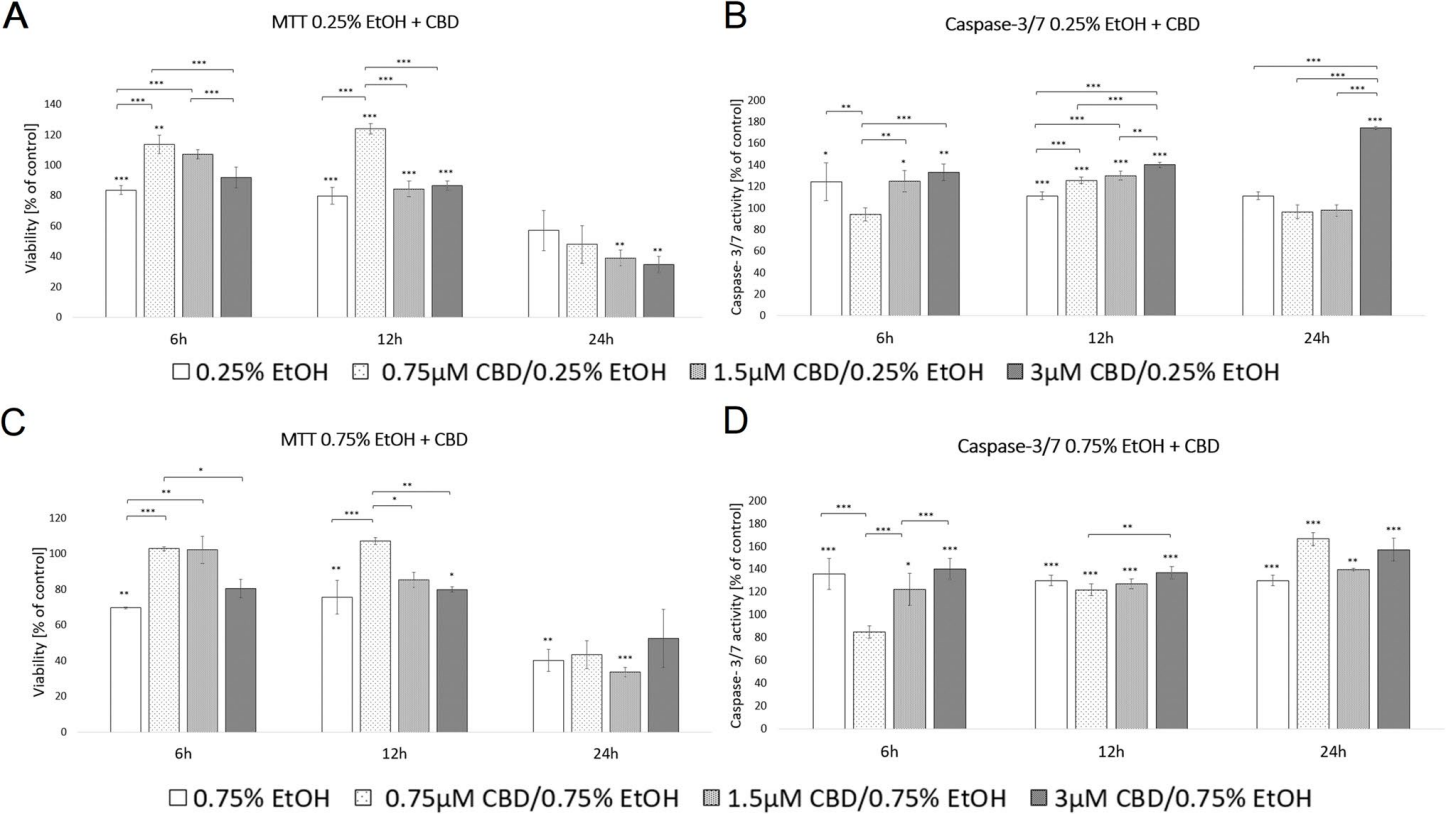
Effects of treatments with different dosages of ethanol and CBD on HDF metabolic activity (**A**, **C**) and apoptosis (**B**, **D**), measured using MTT assay and caspase 3/7 assay over 6 h and 12 h periods. *Statistically significant differences at *p* < 0.05; **statistically significant differences at *p* < 0.01; ***statistically significant differences at *p* < 0.001. Asterisks just above deviation bar show significance relative to control cells (set as 100%)

The two concentrations of EtOH (0.25% or 0.75%) were selected to assess the role of CBD in protecting cells against ethanol-mediated toxic effects. Cells supplemented with 0.75 or 1.5 µM CBD and exposed to 0.25% EtOH for 6 h showed an increase in cell viability by 35.6% (*p* < 0.001) and 28.1% (*p* < 0.001) compared to exposure to EtOH alone, respectively. When cells were incubated for 12 h, the increase in cell survival was noted only at the lower concentration of CBD (*p* < 0.01). When cells were exposed to 0.75% EtOH, similar effects were observed. HDFs supplemented with 0.75 or 1.5 µM CBD and exposed to 0.75% EtOH for 6 h demonstrated an increase in cell viability by 47.6% (*p* < 0.001) and 46.6% (*p* < 0.01), respectively, compared to exposure to EtOH alone. A 12-h incubation period with EtOH and CBD increased the cell viability by 42.1% (*p* < 0.001) only at a concentration of 0.75 µM. HDFs exposed to EtOH and CBD for 24 h did not show any significant difference (Fig. 7; Supplementary File 1).

According to the caspase 3/7 assay, a 6-h period of exposure to 0.25% EtOH and 0.75 µM CBD decreased HDF-cellular caspase 3/7 activity by 24.2% (*p* < 0.01). Furthermore, the luminescence levels after 12 h of exposure to EtOH and CBD were significantly higher in all experimental groups, including 0.75 µM, where an increase in cell viability was noted (*p* < 0.001). When cells were exposed to 0.75% EtOH and supplemented with 0.75 µM CBD for 6 h, a decrease in caspase 3/7 activity (by 37.7%, *p* < 0.001), compared to exposure to EtOH alone, was observed. HDFs exposed to 0.75% EtOH and CBD for 12 and 24 h did not show any significant difference in cellular caspase 3/7 activity (Fig. 7; Supplementary File 1).

### Effect of CBD addition on ethanol-treated cell transcriptomes

**Fig. 8:**
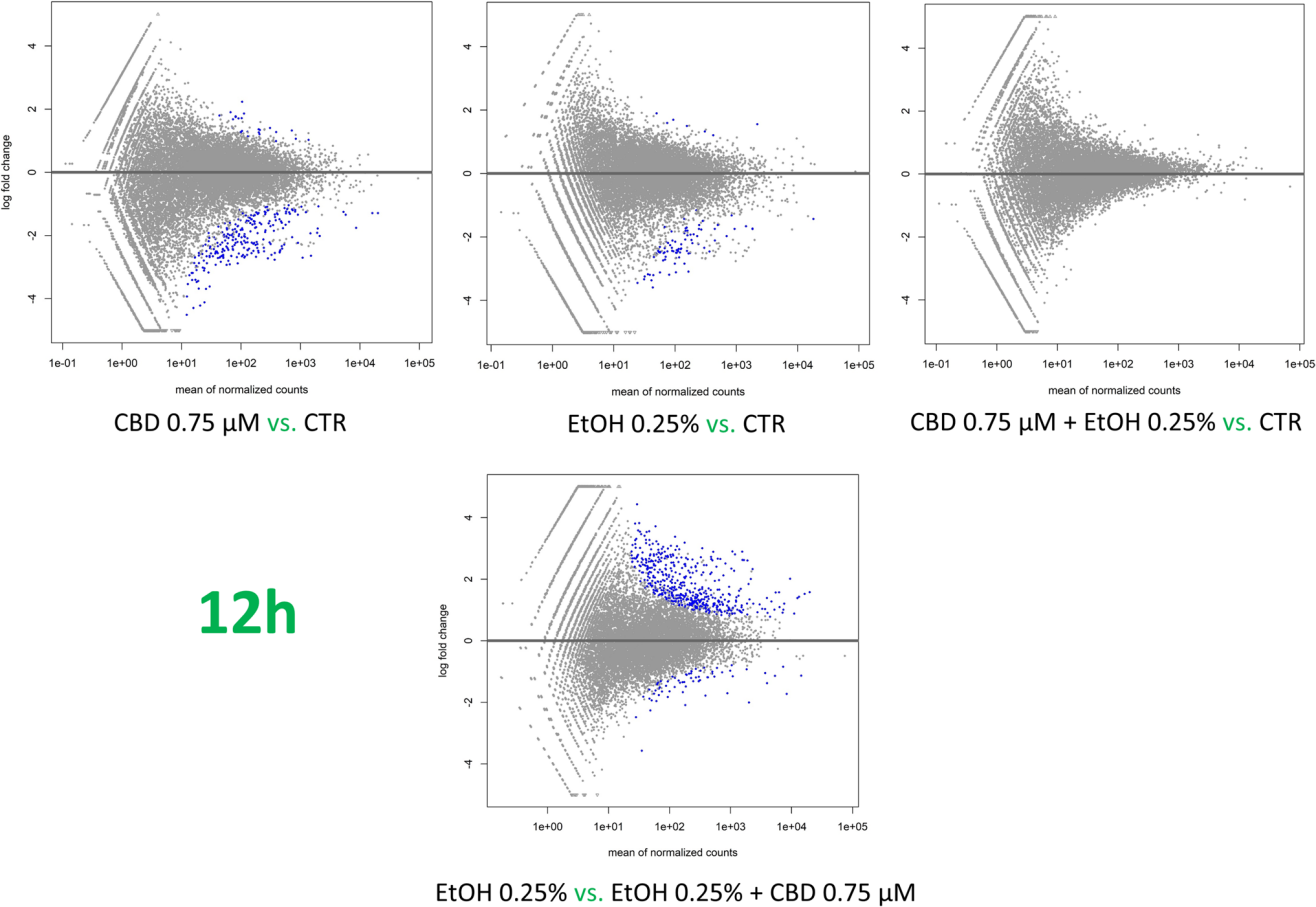
MA plots for changes in transcriptome after CBD 0.75 µM/ethanol 0.25% treatment for 12 h. Each MA plot is designated with the substance, dosage, and comparison group being used in specific comparisons

**Fig. 9:**
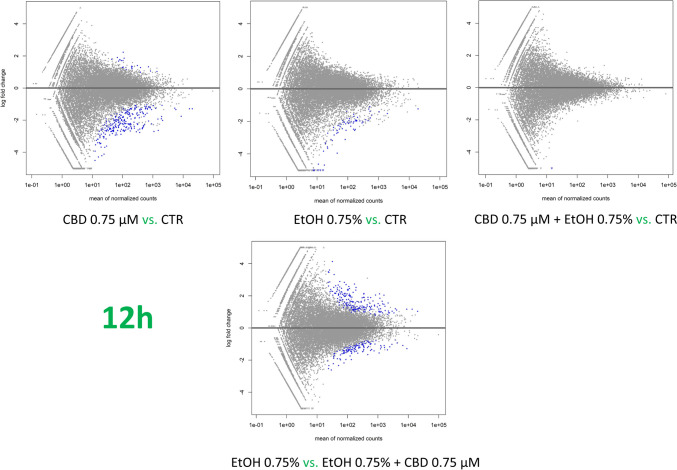
MA plots for changes in transcriptome after CBD 0.75 µM/ethanol 0.75% treatment for 12 h. Each MA plot is designated with substance, dosage, and comparison group used in specific comparisons

**Fig. 10:**
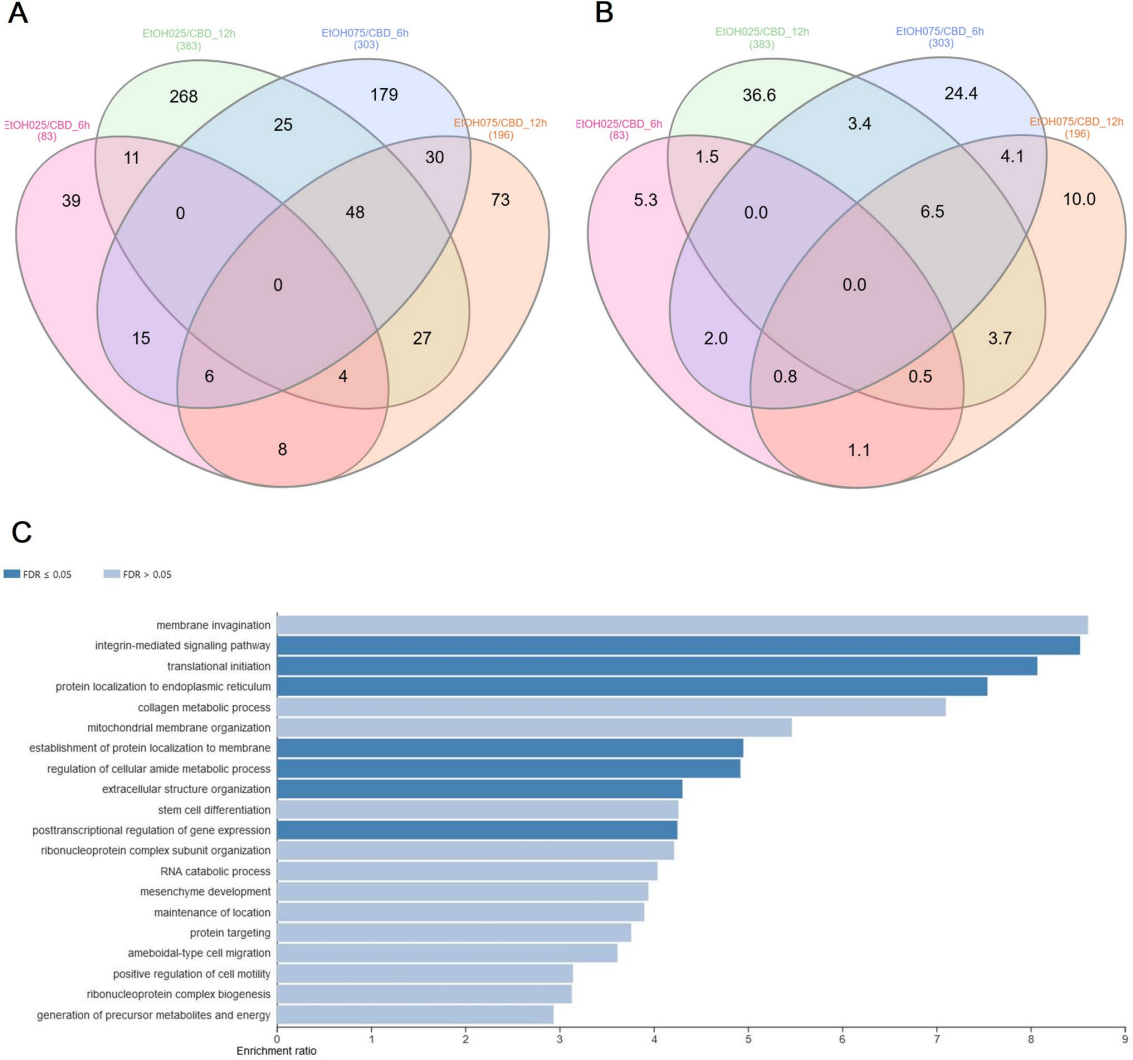
Comparative and functional analysis of genes affected by CBD addition in at least two of the above CBD/ethanol treatments. **A** Venn diagram for genes affected by different CBD/ethanol dosages and times of incubation; **B** Venn diagram for genes affected by different CBD/ethanol dosages and times of incubation—percentage share; **C** overrepresentation analysis (ORA) in GO biological processes for genes common to at least two CBD/ethanol treatments

**Fig. 11:**
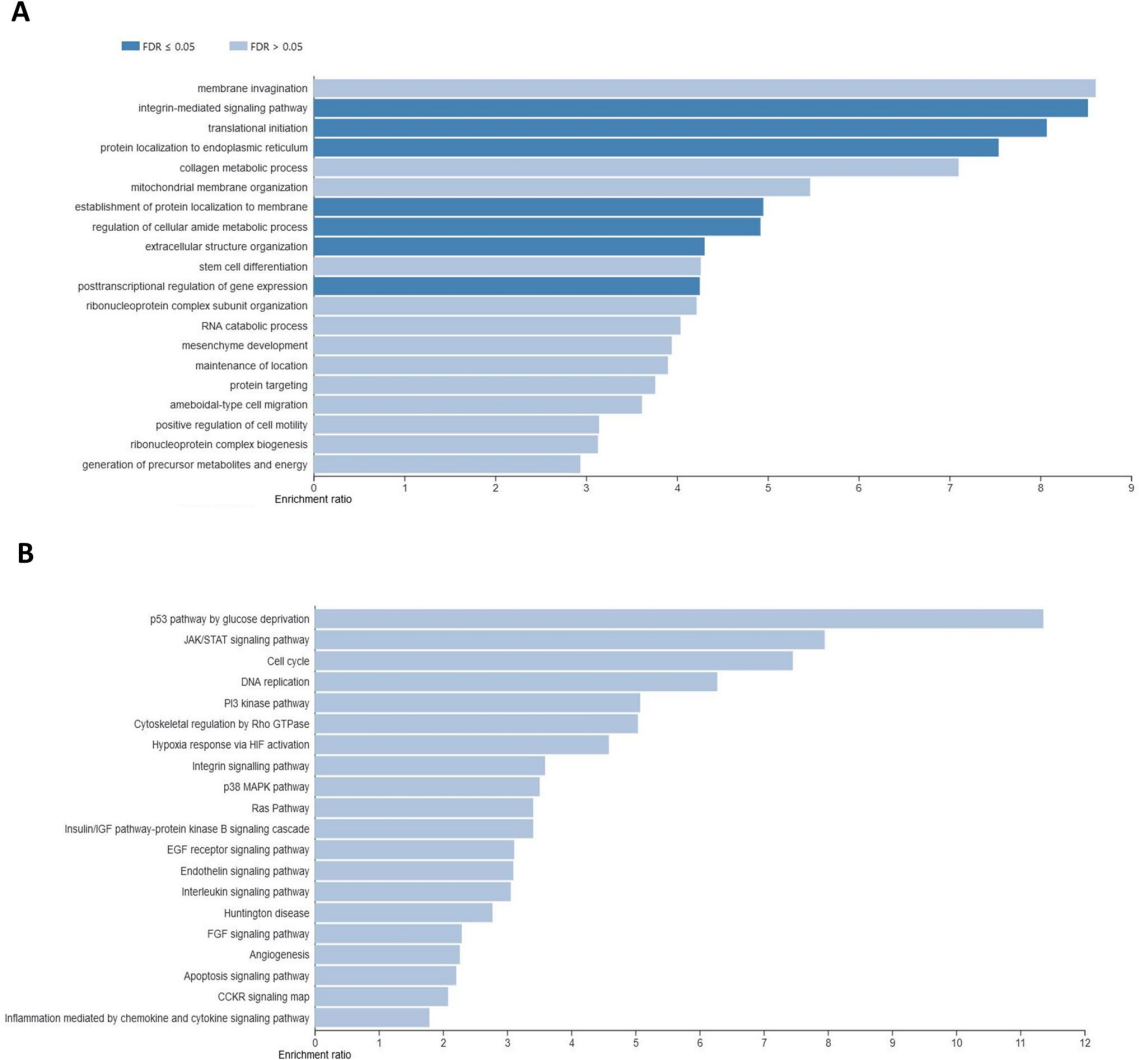
Top 20 enriched biological processes (**A**) and Panther pathways (**B**) (ORA) by genes affected in most of the CBD treatments in ethanol-treated cells

The effect of CBD on ethanol-challenged cells was analyzed separately for cells treated with 0.25% and 0.75% ethanol for 6 and 12 h. As shown in Supplementary File 9, after 6-h treatment, CBD at a concentration of 0.75 µM was able to completely diminish the effect of 0.25% EtOH addition on cultured cells, and no DE genes (FDR > 0.05) were detected in CBD/EtOH treated cells with respect to the control. Similar, but even more effectively exposed effect was observed in the 12-h treatment with the same CBD and EtOH concentrations (Fig. 8). At a higher concentration of EtOH (0.75%), in the 6-h treatment, different expression changes in the profile were observed, and a combination of EtOH and CBD (0.75 µM) resulted in a higher number of altered genes with respect to the control, than for each compound when induced alone. The altered genes (*n* = 188) were mainly downregulated (*n* = 159; 85%) (Supplementary File 10). Nevertheless, the 12-h cells co-treatment with the CBD and EtOH (0.75 µM/0.75%) resulted in the same protective effect of CBD that was observed earlier, which reduced the transcriptional changes stimulated by EtOH (Fig. 9). A functional analysis was performed for non-redundant lists of genes (*n* = 174) that were altered through CBD addition in at least two of the above treatments (Table 5; Fig. 10; Supplementary File 11), specifically in comparisons of cells treated with 0.75% EtOH (as the control) and a CBD 0.75 µM/EtOH 0.75% together (as the treatment). The genes significantly enriched (FDR < 0.05) a biological process associated inter alia with translational initiation, the regulation of the cellular amide metabolic process, the posttranscriptional regulation of gene expression, and the integrin-mediated signaling pathway (Fig. 11; Supplementary File 12). Significant point enrichments were also found for suggested biological processes associated, e.g., with the collagen metabolic process, the positive regulation of cell motility, ameboidal-type cell migration, and stem cell differentiation. Among the enriched Panther pathways (*p* < 0.05), there were the integrin signaling pathway, the p53 pathway via glucose deprivation, and cytoskeletal regulation via Rho GTPase. The top 20 overrepresented categories of the BP and Panther pathways are presented in Fig. 11, and underlying genes can be found in Supplementary File 12.

**Table 5:** Statistics on genes affected by CBD and ethanol treatment (FDR < 0.05) with respect to cells treated with ethanol alone (effect of CBD on ethanol-treated cells)

Treatment	Incubation time (h)	Altered genes (No)	Upregulated (No/%)	Downregulated (No/%)
EtOH 0.25% / CBD 0. 75 µM	6	83	34 (41)	49 (59)
EtOH 0.25% / CBD 0. 75 µM	12	383	346 (90)	37 (9.7)
EtOH 0.75% / CBD 0. 75 µM	6	303	136 (45)	167 (55)
EtOH 0.75% / CBD 0. 75 µM	12	196	125 (64)	71 (36)

### Validation of 3′ mRNA-Seq results via qPCR

A comparison of the expression levels defined with the qPCR and 3′ mRNA-Seq methods of three selected genes (*AKT2*, *COL8A1*, and *PFN1*) showed moderate and significant compliances, as revealed using correlation coefficient analysis. The relations among the applied methods were analyzed for all genes together to evaluate their relative levels of expression. In this analysis, the correlation coefficient among the methods was moderate and highly significant (*r* = 0.465; *p* = 1.2e − 4). This trend was sustained when the expression levels within the samples were averaged into groups, and it showed that the methods correlated positively with *r* = 0.480 and *p* = 0.011 (Supplementary File 13).

## Discussion

The positive effect of CBD in alcohol use disorders (AUDs) has already been documented (Nona et al. 2019) but still needs further proof. It has been shown that CBD is able to reduce steatosis and fibrosis in the liver, and is able to decrease alcohol-related brain damage (De Ternay et al. 2019). Additionally, CBD was shown to interfere with dopamine secretion and D3 receptor mRNA expression (Stark et al. 2020), while D2/3 receptor availability was strongly decreased in high-risk AUD patients (Gleich et al. 2021). However, the exact molecular mechanism, as well as the engaged cell types, pathways, and involved receptors (especially in the fibrosis aspects), is not yet fully recognized. In this study, we attempted to analyze one of the levels at which CBD can act in an organism and affect AUD progression, that is, the transcriptomic responses of fibroblasts to alcohol toxicity. This response can be particularly significant for alcohol-stimulated liver disease with fibrosis, which involves the excessive accumulation of extracellular matrix proteins containing collagen (produced by different cell types, including fibroblasts) and chronic inflammatory processes (Bataller and Brenner 2005). The cellular model applied here can also be useful for describing the general effect of CBD on ethanol-exposed cells, as fibroblasts, similarly to hepatic cells, are able to metabolize ethanol (Petersen et al. 1980), and thus, they also bear the burden of ethanol toxicity in AUD-affected patients. By taking advantage of next-generation sequencing, we assumed that we would be able to picture the transcriptomic responses of fibroblasts to ethanol challenge, as well as CBD treatment, which should presumably alleviate ethanol-induced transcriptome alterations.

Our study hypothesis was largely confirmed. We found that CBD at the lowest applied concentration (0.75 µM) was able to stimulate depressed metabolism (viability) and to reduce the level of apoptosis of cells challenged with different concentrations of ethanol to the level observed in the control cells. However, at higher CBD concentrations, this effect was weaker or even reversed at the highest concentrations and the longest incubation times. Similar observations were made at the transcriptome level, in which cells treated with ethanol (at both doses) and CBD (0.75 µM) had similar expression profiles to the control cells (unlike cells treated with ethanol alone). Similar observations were previously made on gingival fibroblast cells, in which lower concentrations of CBD (< 1 µM) increased the expression of transforming growth factor (*TGF-β*) and metalloproteinase (MMPs) genes, while higher concentrations had reversed effects on the expression of these genes (Rawal et al. 2012). The dosage-dependent effect of CBD on cells’ viability and apoptosis was also reported in several other studies with different experimental setups (Pagano et al. 2020). The observation that CBD is able to counteract ethanol-induced transcriptome changes in a dose-dependent manner is the most interesting aspect of this study, as it may have potential implications for CBD-including AUD therapies. First, it seems that CBD at low concentrations exerts an unrecognized effect that protects cells from transcriptional alterations caused by the presence of ethanol in their immediate environment. Second, the cells that are challenged with ethanol react differently to CBD, and they do not respond with transcriptome changes, compared to when they are supplemented separately. The ability of CBD to counteract the effects of ethanol in fibroblasts may be especially interesting when considering the fact that fibroblasts (or cells of the myofibroblast phenotype) are key contributors to fibrotic extracellular matrix accumulation during liver fibrosis (Bataller and Brenner 2005; Zeisberg et al. 2007). In the course of fibrosis, the chronic injury of hepatocytes causes the activation of collagen type I-producing myofibroblasts that produce fibrous scars in the liver. Our results from transcriptome analysis showed that CBD treatment causes changes in the expression of several genes in human fibroblasts, associated with collagen-containing extracellular matrix formation. Among the genes (that were affected by at least three different CBD treatments), CBD downregulated the expression of, e.g., the *BGN* gene that encodes Biglycan, which is a secreted proteoglycan that is involved in collagen fibril assembly, while its fragmentation is likely to be associated with collagen turnover during the pathogenesis of diseases that involve dysregulated extracellular matrix remodeling (ECMR), such as liver fibrosis (Ciftciler et al. 2017). The other downregulated gene encodes collagen type I alpha 1 chain (*COL1A1*), which is the major component of type I collagen (Ma et al. 2019), a main constituent of ECM in liver fibrosis. The downregulation of other collagen-type subunits was also observed, which included, e.g., collagen type VIII alpha 1 chain (*COL8A1*), collagen type VIII alpha 2 chain (*COL8A2*), and collagen type V alpha 2 chain (COL5A2). It was shown that a lack of collagen VIII reduces myofibroblast differentiation and fibrosis in mouse hearts (Skrbic et al. 2015), and that *COL8A*1 and *COL5A2* are dysregulated in progressing liver fibrosis in rodents (Fagone et al. 2015; Kasai et al. 2021). Del Río et al. (2022) examined the anti-fibrotic effects of CBD in the skin using NIH-3T3 fibroblasts and HDF (an in vitro model), as well as the bleomycin (BLM)–induced mouse model of skin fibrosis (an in vivo model). NIH-3T3 cells transiently transfected with COL1A2-luc plasmid and pretreated with CBD demonstrated a significant inhibition of TGFβ1 stimulation on COL1A2 transcription that confirmed that CBD blunted the effects of fibrogenic stimuli on cultured fibroblasts. Moreover, both the intraperitoneal and oral administration of CBD stimulated potent anti-inflammatory and anti-fibrotic activities in BLB-induced dermal fibrosis in vivo (Del Río et al. 2022).

While searching for other genes altered by CBD treatment with links to the fibrosis process, we found *BMP4*, which was downregulated in different CBD treatments and codes for bone morphogenetic protein 4, which is a member of the transforming growth factor-beta (*TGF-β*) superfamily. It was shown that members of this family were reversely correlated with *TGF-β1* expression in human and mouse fibrotic livers. Exemplarily, the mechanistic study revealed that *BMP2* inhibits TGF-β1-induced hepatic stellate cells (HSCs) activation associated with the attenuated expression of α-smooth muscle actin (α-SMA) and fibronectin, and reversed epithelial-to-mesenchymal transition markers, indicating that BMP2 serves as a protective agent in liver fibrosis (Chung et al. 2018). It was also displayed that *BMP4* alleviates hepatic steatosis by increasing hepatic lipid turnover and inhibiting the mTORC1 signaling axis in hepatocytes (Peng et al. 2019).

Some other strong candidate genes for CBD action with connotations to fibrosis were also detected in the RNA-Seq analysis performed, and included *PLD2*—its family can be linked with a decrease in type I collagen levels in hepatic stellate cells via the induction of autophagy (Chang et al. 2020; Seo et al. 2014), *IGF1R*, which is associated with the reduction of fibrosis and the amelioration of general liver function by IGF1 (Adamek and Kasprzak 2018; Wang et al. 2023), and *HMGB2*, the overexpression of which initiates fibrogenesis and augments the effect of CCl4 in inducting fibrosis in mice (Huang et al. 2017). Del Río et al. demonstrated for the first time the efficacy of CBD in reducing CCL4-induced hepatic fibrosis. In this non-alcoholic liver fibrosis mouse model, CCl4 treatment significantly induced the accumulation of collagen in the liver in contrast to the healthy group, and CBD substantially reduced the fibrotic liver area (Del Río et al. 2022). The fact that CBD is able to downregulate several profibrotic and ECM-related genes may have important implications in liver fibrosis and in AUD generally; however, this finding certainly needs further investigation.

Another important finding of this study is that CBD is able to affect cell viability and apoptosis in ethanol-challenged fibroblast cells. The effect of CBD on cell viability was previously described. The authors found a mainly cytotoxic (but dosage-dependent) effect of CBD on cells in the MTT assay; however, the studies mostly considered high CBD concentrations or longer incubation times (24 h or longer), compared to this study (Alves et al. 2021; Cerretani et al. 2020; Motadi et al. 2023). Our results demonstrated that the effects of CBD on cell viability are different when considering low dosages and short incubation times. At shorter times (6 and 12 h) and at lower concentrations, CBD was able to stimulate cell viability; however, at higher CBD concentrations and with a 24-h incubation time, CBD reduced cell viability by up to 58% of the control. In the case of caspase 3 and 7 activities, CBD alone stimulated their activities, which were raised together with the increase in the CBD concentration. Nevertheless, in ethanol-treated cells, the lowest concentration of CBD was able to reduce the level of apoptosis to 84% of the control. This effect was not permanent and ceased after 12 and 24 h of incubation with the same CBD concentration. In previous studies, the effect of CBD on cell apoptosis was clearly established, and was mainly proapoptotic in various cells (mainly cancer cells) and experimental setups (Gross et al. 2021; Hamad and Olsen 2021; Shrivastava et al. 2011), which was concordant with most of our observations. This might be especially important when considering a basic study on hematopoietic stem cells (HSCs), which revealed that CBD induced the downstream activation of the IRE1/ASK1/c-Jun N-terminal kinase pathway that promoted apoptosis, leading to HSC death. The authors suggested that CBD can be used as a potential therapeutic agent for chronic hepatitis that leads to liver fibrosis by selectively inducing the apoptosis of activated HSCs (Lim et al. 2011).

While critically evaluating our results, as the limiting factors of this study, we point toward missing sequencing results for some treatments in the RNA-Seq analysis. To avoid a batch effect, we decided not to include reproduced sequencing results in the analysis. Nevertheless, the general findings of this study are based on several comparisons and various treatments (times and doses) that together provide sufficient amounts of replicates to draw credible conclusions. Another drawback of this study is that the human dermal fibroblasts used are not a perfect model for, e.g., hepatic myofibroblasts, to which the functions of most of the above-described processes were related. However, these two cell phenotypes are largely similar, and interestingly, it was shown that cultured fibroblasts retain fibroblast markers only in early passage cultures, but with an increase in cell passages, they begin to transdifferentiate into myofibroblasts (Santiago et al. 2010). Fibroblasts, regardless of their origin (dermal or hepatic), share fundamental roles in the synthesis of extracellular matrix (ECM) components and tissue repair. Both dermal and hepatic fibroblasts respond to fibrogenic stimuli such as TGF-β, leading to increased production of collagen and other ECM proteins. The core fibrotic response—activation of fibroblasts into myofibroblasts—can be effectively studied in HDFs because they exhibit similar signaling pathways involved in fibrosis. Human dermal fibroblasts can be used as cellular models in various disease studies because they are readily accessible and do not require complex genetic manipulation. These fibroblasts respond to fibrogenic stimuli of TGF-β, a key driver of fibrosis in many tissues, including the liver (Mesdom et al. 2020). Several cellular processes in both mentioned cell types should remain the same, and thus fibroblasts should be an acceptable initial model for liver myofibroblasts. Notwithstanding, further studies are required, and caution should be exercised when extending findings from in vitro to in vivo applications, particularly concerning the impact of CBD on fibrosis. Presented findings require additional support, including the use of more direct models of liver fibrosis.

In conclusion, our study demonstrated the time- and dosage-dependent aspects of CBD actions on HDF (both treated with ethanol and untreated) viability and metabolism, and its transcriptome profile. In the case of CBD, the lowest applied dosage (0.75 µM) had the most desired effect on cell viability and apoptosis, especially when cells were challenged with ethanol. However, this dosage had only minor effects on the cell transcriptome under short-term treatment, and mainly downregulated gene expression at 12 h of incubation. CBD treatment altered the expression levels of several genes that are related to extracellular matrix biogenesis and regulation that could be in perspective associated, e.g., with a fibrosis process in the liver that is a common health problem in AUD. Our result also suggests that lower CBD doses may be more beneficial for anticipated therapeutic purposes in AUD, and that their effects should be more carefully analyzed in clinical studies.

## Supplementary Information

Below is the link to the electronic supplementary material.

Supplementary file1 (XLS 71 KB)

Supplementary file2 (XLSX 15 KB)

Supplementary file3 (XLSX 24221 KB)

Supplementary file4 (XLSX 6170 KB)

Supplementary file5 (XLSX 711 KB)

Supplementary file6 (XLSX 21843 KB)

Supplementary file7 (XLSX 12 KB)

Supplementary file8 (XLSX 319 KB)

Supplementary file9 (PNG 1533 KB)

Supplementary file10 (PNG 1637 KB)

Supplementary file11 (XLSX 23322 KB)

Supplementary file12 (XLSX 425 KB)

Supplementary file13 (XLSX 8 KB)

## Data Availability

Raw sequencing reads and read counts within genes were deposited in the Gene Expression Omnibus (GEO) and Sequence Read Archive (SRA) databases from the National Center for Biotechnology Information (NCBI) under the accession number GSE232624.

## Abbreviations

AUD: Alcohol use disorder
CBD: Cannabidiol
EtOH: Ethanol
HDFs: Human dermal fibroblasts
MTT: 3-(4,5-Dimethylthiazol-2-yl)-2,5-diphenyltetrazolium bromide
PCA: Principal component analysis
RNA-Seq: RNA sequencing
THC: Tetrahydrocannabinol
