# Supplementary figures and images for: Cannabidiol (CBD) modulates the transcriptional profile of ethanol-exposed human dermal fibroblast cells
Source: J Appl Genet. 2024 Oct 28;65(4):773–96. doi: 10.1007/s13353-024-00915-7 (PMC11561130; doi:10.1007/s13353-024-00915-7)

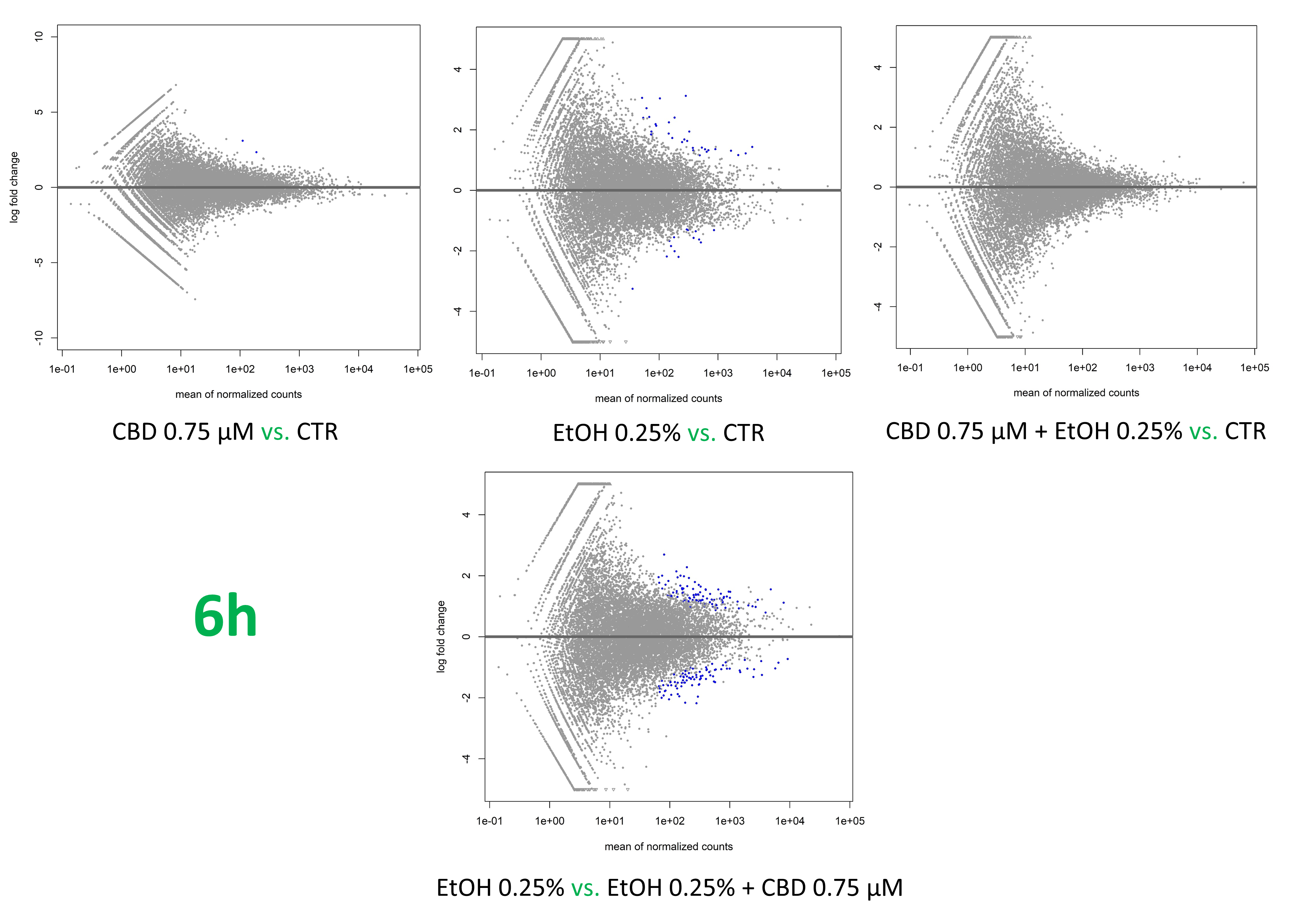

Supplement: Supplementary file 9 — Supplementary file9 (PNG 1533 KB) [file 13353_2024_915_MOESM9_ESM.png]

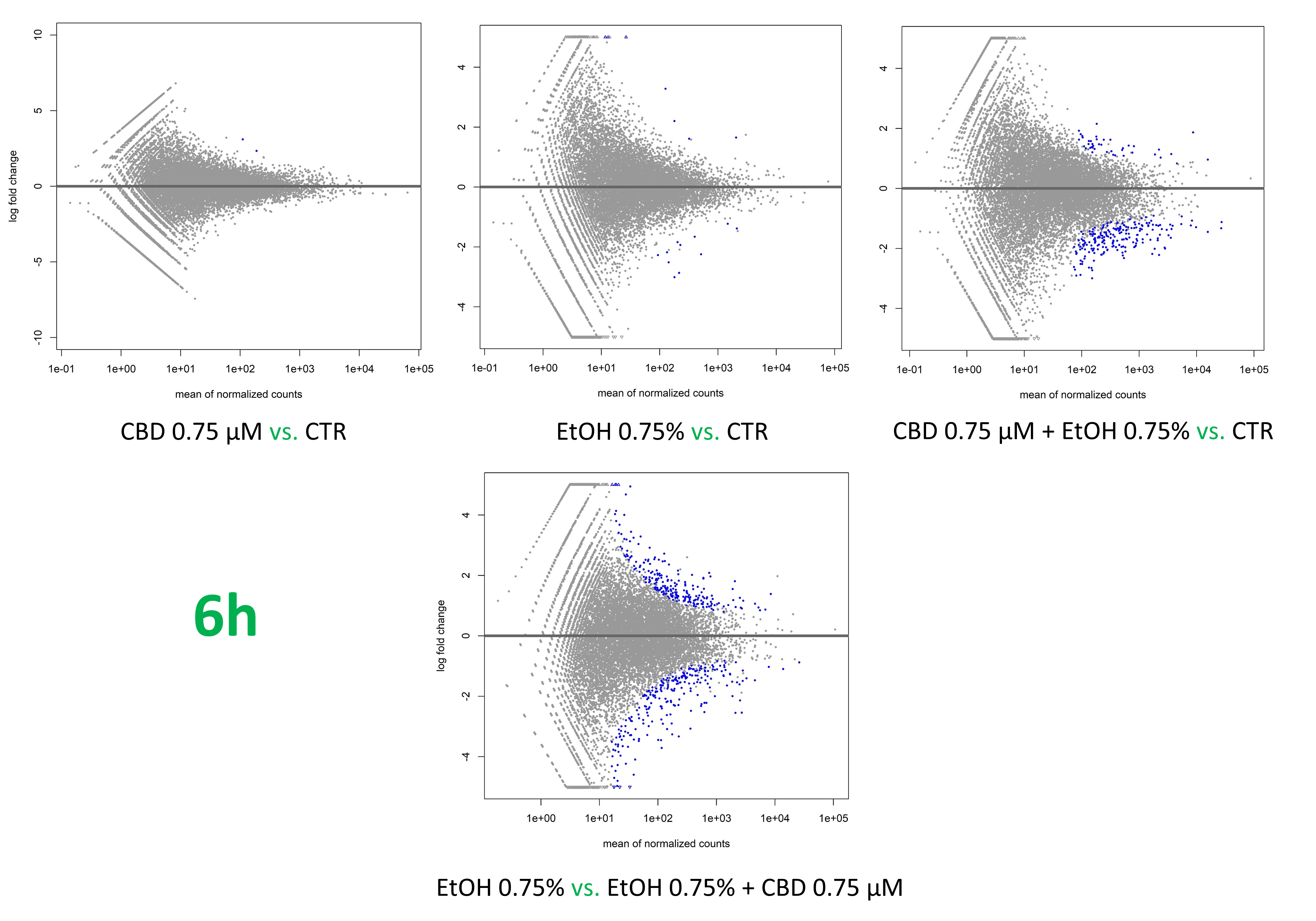

Supplement: Supplementary file 10 — Supplementary file10 (PNG 1637 KB) [file 13353_2024_915_MOESM10_ESM.png]
